# Radiotherapy in Combination with Systemic Therapy for Multiple Myeloma—A Critical Toxicity Evaluation in the Modern Treatment Era

**DOI:** 10.3390/cancers15112909

**Published:** 2023-05-25

**Authors:** Michael Oertel, Tom Schlusemann, Evgenii Shumilov, Gabriele Reinartz, Anne Bremer, Stephan Rehn, Georg Lenz, Cyrus Khandanpour, Hans Theodor Eich

**Affiliations:** 1 Department of Radiation Oncology, University Hospital Muenster, Albert-Schweitzer-Campus 1, 48149 Muenster, Germany; t_schl42@uni-muenster.de (T.S.); gabriele.reinartz@ukmuenster.de (G.R.); stephan.rehn@ukmuenster.de (S.R.); hans.eich@ukmuenster.de (H.T.E.); 2 Department of Medicine A, Hematology, Oncology and Pneumology, University Hospital Muenster, Albert-Schweitzer-Campus 1, 48149 Muenster, Germany; evgenii.shumilov@ukmuenster.de (E.S.); georg.lenz@ukmuenster.de (G.L.); 3 Department of Oncology, St. Franziskus-Hospital, Hohenzollernring 70, 48145 Muenster, Germany; anne.bremer@sfh-muenster.de; 4 Department of Hematology and Oncology, University Hospital of Schleswig-Holstein, University of Luebeck, Ratzeburger Allee 160, 23538 Luebeck, Germany; cyrus.khandanpour@uksh.de

**Keywords:** multiple myeloma, radiation therapy, combined modality treatment, safety, side effects

## Abstract

**Simple Summary:**

Radiotherapy is essential for the management of symptomatic osteolytic lesions in multiple myeloma but usually requires a combination with systemic therapy. This study analyzes the acute toxicities of radiotherapy in this setting and demonstrates the feasibility of modern combined modality treatments without significant increases in hematological and non-hematological side effects. However, high-grade leukocytopenia is more frequent following radiotherapy when systemic therapy is given simultaneously. Treatment of five bones or more was associated with a significant increase in thrombocytopenia and leukocytopenia during radiotherapy.

**Abstract:**

Radiotherapy (RT) is an established treatment modality in the management of patients with multiple myeloma (MM), aiming at analgesia and stabilization of osteolytic lesions. As a multifocal disease, the combined use of RT, systemic chemotherapy, and targeted therapy (ST) is pivotal to achieve better disease control. However, adding RT to ST may lead to increased toxicity. The aim of this study was to evaluate the tolerability of ST given concurrently with RT. Overall, 82 patients treated at our hematological center with a median follow-up of 60 months from initial diagnosis and 46.5 months from the start of RT were evaluated retrospectively. Toxicities were recorded from 30 days before RT up to 90 days after RT. 54 patients (65.9%) developed at least one non-hematological toxicity, with 50 patients (61.0%) showing low-grade (grade I or II) and 14 patients (17.1%) revealing high-grade (grade III and IV) toxicities. Hematological toxicities were documented in 50 patients (61.0%) before RT, 60 patients (73.2%) during RT, and 67 patients (81.7%) following RT. After RT, patients who had received ST during RT showed a significant increase in high-grade hematological toxicities (*p* = 0.018). In summary, RT can be safely implemented into modern treatment regimens for MM, but stringent monitoring of potential toxicities even after completion of RT has to be ensured.

## 1. Introduction

Multiple myeloma (MM) belongs to the group of plasma cell neoplasms and is characterized by the monoclonal proliferation of malignant plasma cells in the bone marrow and the secretion of monoclonal immunoglobulins [1]. In recent decades, the incidence of MM has increased significantly, with an expected number of 33,500 new cases and 14,100 myeloma-related deaths in the US in 2022 [2,3]. In Germany, the incidence has increased by about 20% since 1999, without relevant changes in age-standardized rates [4]. Osteolytic lesions are a hallmark of MM, occurring in more than 60% of patients at first diagnosis, and are often associated with bone pain and pathological fractures [5]. Radiotherapy (RT) targets extramedullary and osteolytic lesions, enabling local control with the goal of analgesia and stability [6,7]. In contrast to solitary osseous or extramedullary plasmacytomas, which may be addressed with RT alone [8,9], MM is a multifocal, systemic disease, and combinations of RT with systemic therapy (ST) are essential. The latter encompasses various categories, such as conventional chemotherapy and immunomodulatory agents, but also novel modalities like proteasome inhibitors, (bispecific) antibodies, and chimeric antigen receptor T-cells [10,11]. The evolution and intensification of modern myeloma medication cause concerns about the concurrent use of RT regarding safety and efficacy, with some previous works postulating a continuation or interruption/postponement of RT, respectively [12,13,14]. This is of pivotal importance, as RT is considered an integral part of treatment strategies for MM even in cases of critical shortage of resources, e.g., during the COVID-19 pandemic [15,16]. Previous retrospective analyses point towards a safe combination of RT and ST, but patient numbers are often limited or evaluations were conducted prior to the introduction of modern therapeutic agents [17,18,19,20].

Consequently, there is an unmet need for a decisive analysis of combined modality treatment in the modern treatment era of MM. Our analysis investigates the use of RT as part of multimodal regimens and provides a detailed evaluation of acute toxicities, covering both hematological and non-hematological side effects. A critical appraisal of modern RT strategies is provided, as is a discussion covering relevant preceding publications on multi-modality treatment in MM.

## 2. Materials and Methods

### 2.1. Clinical Data

The current study was conceptualized as a monocentric retrospective analysis and approved by the respective institutional review board (Ethikkommission der Ärztekammer Westfalen-Lippe; protocol code 2020-873-f-S; 11 December 2020). Overall, 96 patients treated with RT alone or in combination with ST at our hematological center between 1999 and 2019 with a minimum follow-up of three months were analyzed. Of these, 14 patients were excluded from the analysis due to radiation of a solitary osseous plasmacytoma (7) or due to insufficient follow-up data (7). Clinical data and patient characteristics were extracted from the hospital information system (Orbis, Dedalus Health Care, Bonn, Germany), including documentation of toxicities, doctors’ letters, and treatment plans. Details on RT were provided by the RT planning system (Aria, Varian Medical Systems, Pao Alto). Acute toxicities were recorded for a period from 30 days before the start of RT to 90 days after completion and classified using the National Cancer Institute’s Common Terminology Criteria for Adverse Events version 5.0 (CTCAE) [21]. Non-hematological toxicities were graded according to the documentation of the treating physicians. Hematological toxicities were graded separately based on available laboratory values, with an evaluation of the last laboratory value before RT, the lowest value during RT, and the lowest value up to 90 days after RT. In cases of insufficient data, general practitioners and radiation oncologists in private practices were contacted to provide further information. The data collection ended in October 2022. Only the first myeloma-related radiation was included in the analysis.

### 2.2. Statistical Analysis

All statistical analyses were performed using SPSS^®^ version 28.0 (IBM^®^, Armonk, NY, USA). For inferential statistics, the patient collective was divided into a group with combined RT and ST (RT/ST group) and RT alone (RT group), respectively. For inclusion in the RT/ST group, at least one dose of ST had to be given between the start and end of RT. Inferential statistics were performed for binary variables with Fisher’s exact test and for categorical characteristics with the chi-square test (χ2). A *p*-value of <0.05 was set as the significance level, and no adjustment was made for multiple testing. For categories with fewer than five expressions, no adjustment was made in the chi-square test.

## 3. Results

A total of 82 patients with a median age of 58.5 years at initial diagnosis (39–85 years) were included in the study, 50 of whom were male (61%) and 32 female (39%; see Table 1 for details). The median time between the initial diagnosis and the start of RT was one month (0–86 months). The median follow-up from the initial diagnosis was 60 months (5–183 months) and 46.5 months (4–170 months) from the start of RT. According to the Reversed International Staging System (R-ISS) [22], 17 patients (20.7%) were classified as stage I, 44 as stage II (53.7%), and 5 as stage III (6.1%) at initial diagnosis. The majority of patients had an ECOG performance status [23] of 0 (14 patients, 17.1%) or 1 (27 patients, 32.9%). Osseous lesions were located in the spine in 80 patients (97.6%), in the pelvis in 58 (70.7%), in the extremities in 31 (37.8%), in the skull in 32 (39.0%), in the ribcage in 35 (42.7%), and in the shoulders in 17 patients (20.7%).

Overall, 36 patients (43.9%) underwent at least one myeloma-related surgical procedure prior to RT, predominantly biopsy (17 patients, 47.2%) or osteosynthesis (18 patients, 50.0%; see Table 2 for details). Regarding ST, 76 patients (92.7%) obtained systemic treatment within one month prior and up to three months after RT. In detail, 39 patients (47.6%) received ST in the month before RT, 53 patients (64.6%) during RT, and 75 patients (91.5%) after RT. Stem cell mobilization was performed in 13 patients (15.9%), and autologous stem cell transplantation took place in eight patients (9.8%) following RT. Treatment consisted of at least one novel agent in 70 patients (85.4%), with 56 patients (68.3%) and 32 patients (39.0%) undergoing treatment with proteasome inhibitors or immunomodulatory imide drugs, respectively. Furthermore, 72 patients (87.8%) were treated with glucocorticoids, 35 patients (42.7%) with alkylating agents, and 16 patients (19.5%) with topoisomerase inhibitors. Other agents were used in four patients (4.9%). In 68 patients (82.9%), osteoprotective treatment was ensured by bisphosphonates or denosumab.

A total of 134 localizations were irradiated, with each patient being irradiated at a median of one site (1–4; see Table 3 for details). The median radiation dose was 40 Gray (Gy; 8–59 Gy). The majority of lesions were targeted using intensity-modulated RT (IMRT) (62 sites, 46.3%) or three-dimensional conformal RT (3D-CRT) (54 sites, 40.3%). The most frequently irradiated sites were the spine (95 sites, 70.9%) and the pelvis (34 sites, 25.4%).

Fifty-four patients (65.9%) developed at least one non-hematological toxicity, with no significant difference between the RT group and the RT/ST group (65.5% vs. 66.0%, *p* = 1.00; see Table 4 for details). Low-grade non-hematological toxicities (grade I or II) occurred in 50 patients (61%), in comparison to 14 patients (17.1%) with high-grade toxicities (grade III or IV). No toxicity-related deaths were observed. The most common non-hematological toxicities were cutaneous (32.9% vs. 1.2% for low-grade vs. high-grade toxicities, respectively), fatigue (22.0% vs. 1.2%), mucosal (13.4% vs. 4.9%), and gastrointestinal toxicities (13.4% vs. 3.7%), with no significant difference in frequency between the RT and RT/ST groups.

**Figure 1:**
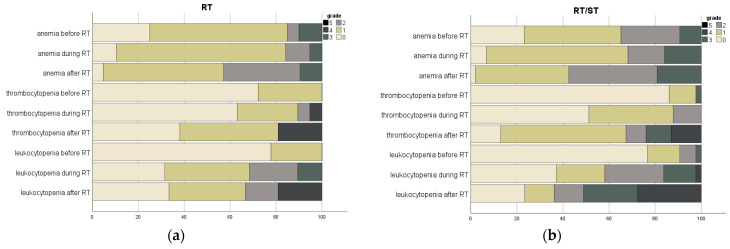
Hematological toxicities subdivided into 30 days before radiotherapy (RT), during RT, and up to 90 days after RT. For analysis, the last available laboratory results before RT were used, compared to the highest measured toxicity during and after RT. Evaluation is divided into the RT group (**a**), receiving monomodal treatment with RT-only, and the RT/ST group (**b**), treated with concurrent systemic therapy.

All patients with documented laboratory values developed at least one hematological toxicity (73/82 patients, 89%; see Table 5, Table S1 and Figure 1 for more details). Of these, 37 patients (45.1%) also suffered from at least one high-grade toxicity (grade III or IV). There was no significant difference in overall high-grade toxicities between the RT and RT/ST groups (27.6% vs. 54.7%; *p* = 0.349). While 61.0% of patients (50 patients) already showed at least one hematological toxicity in the period of 30 days before RT, this proportion increased to 73.2% (60 patients) during RT and 81.7% (67 patients) after RT. High-grade toxicities were seen in seven patients (8.5%) before RT, 16 patients (19.5%) during RT, and 31 patients (37.8%) after RT. Despite no significant difference in high-grade toxicities between the RT and RT/ST groups before and during RT (before RT: 6.9% vs. 9.4%, *p* = 1.00; during RT: 13.8% vs. 22.6%, *p* = 0.754), a significant increase in high-grade toxicities was observed in the combined-modality group (17.2% vs. 49.1%, *p* = 0.018) after RT.

**Figure 2:**
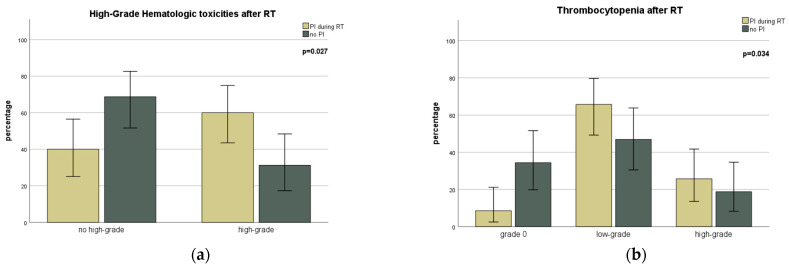

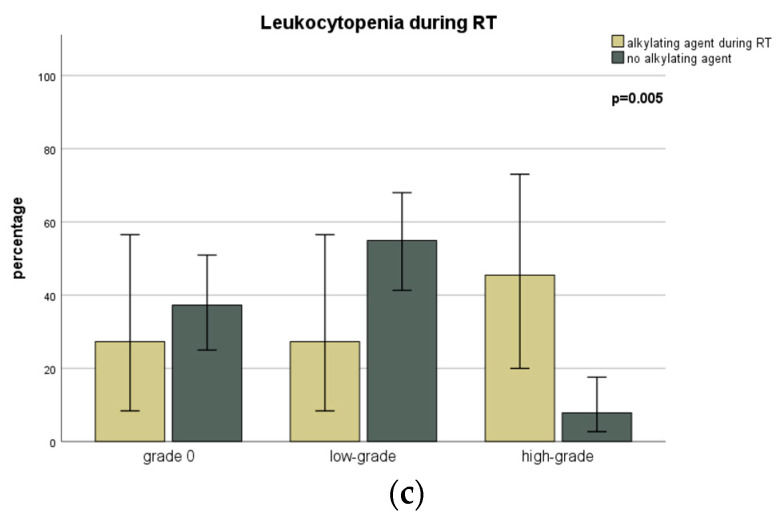
Bar graph representation of hematological toxicities depending on the type of systemic therapy concurrent with radiotherapy (RT). Grade of toxicities (**a**) and thrombocytopenia after RT (**b**) for the patient group treated with proteasome inhibitors (PI) in comparison to patients without. (**c**) Grade and percentage of leukocytopenia during RT depending on the use of alkylating agents. Error bars present 95% confidence intervals.

Hematological and non-hematological toxicities were analyzed for each substance class if data on at least 10 patients was available. No decisive analysis of the use of glucocorticoids was performed, as all patients treated with simultaneous ST also received a glucocorticoid (see Table 4 and Table 5 for details). The concurrent use of proteasome inhibitors showed a significant increase in high-grade hematological toxicities after the end of RT (*p* = 0.027; Figure 2a), especially regarding thrombocytopenia (*p* = 0.034; Figure 2b). With the use of alkylating agents, there was a significant increase in leukocytopenia during radiotherapy (*p* = 0.005; Figure 2c). A further significant increase in hematological or non-hematological toxicities during or after RT could not be shown for the different substance classes.

**Figure 3:**
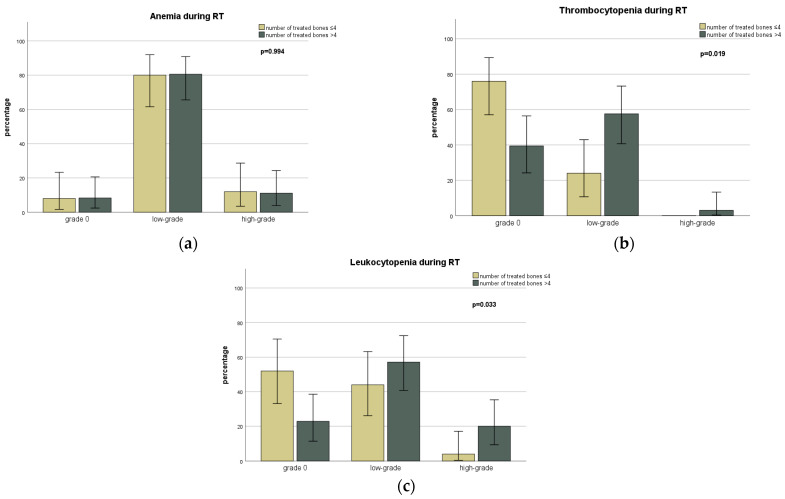
Bar graphs representation of the severity of anemia (**a**), thrombocytopenia (**b**), and leukocytopenia (**c**) during radiotherapy in relation to the number of bones irradiated. Error bars present 95% confidence intervals.

Dichotomous stratification between <40 Gy and ≥40 Gy revealed an increase in low-grade non-hematological toxicities in the ≥40 Gy group (*p* = 0.004), with no dose-side-effect relationship for high-grade non-hematological toxicities or hematological (low- and high-grade) toxicities. Concerning the number of bones irradiated, it could be shown that simultaneous irradiation of five or more bones led to a significantly higher rate of thrombocytopenia (*p* = 0.019) and leukocytopenia (*p* = 0.033) during RT (see Figure 3 for details). This effect was not seen regarding the rate of anemia (*p* = 0.994).

## 4. Discussion

The presented analysis outlines important data on the use of RT as a treatment element for MM in the modern era. It emphasizes that (1) RT is a tolerable and efficient treatment for MM with a low rate of high-grade toxicities; (2) combined-modality treatment may be established safely in most cases; (3) an increase in high-grade hematological toxicities after RT, especially leukocytopenia, was shown for the combined modality group requiring careful laboratory monitoring; (4) large RT fields encompassing five or more bones lead to an increase in leukocytopenia and thrombocytopenia and demand a carefully considered indication.

Overall, both hematological and non-hematological toxicities are common side effects of treatment in MM. Hematological toxicities may be caused not only by MM itself but also by treatment modalities. Depending on the treatment regimen and indication (induction, consolidation, or maintenance), high-grade hematological toxicities were observed in 30% up to 94.9% of patients in the literature [24,25,26,27,28,29,30,31]. Attal et al. described particularly high values for an induction therapy consisting of lenalidomide, bortezomib, and dexamethasone, followed by stem cell therapy and one-year lenalidomide maintenance therapy (94.9%) [24].

This raises the question of whether RT leads to a further increase in toxicities, which has been discussed in the literature with unclear results [17,18,19,20]. Evidence is sparse, with some analyses lacking control groups [20] or only investigating typical radiotherapy-associated toxicities without consideration of potential aggravation of (hematological) toxicities due to systemic therapy [19].

In this regard, all patients in our analysis with documented laboratory values developed at least one low-grade hematological toxicity until 90 days after completion of RT. Concerning high-grade hematological toxicities, 45.1% of patients were affected, with a steady increase over time (8.5% before RT up to 37.8% after RT). This is mirrored by a continued rise in the use of ST (47.6% before RT vs. 64.6% during and 91.5% after RT). While no significant difference in the degree of hematological toxicity could be demonstrated between the RT and RT/ST groups during RT, there was a significant increase in high-grade toxicities in the RT/ST group after the completion of RT.

Non-hematological toxicities were low-grade in most cases, with only a small proportion of patients revealing grade III or IV toxicities without any grade V toxicity up to 90 days after RT. The most common non-hematological adverse events were dermatitis, fatigue, nausea, and gastrointestinal and mucosal toxicities, consistent with previous studies [17,18,19,20] (see Table 6 for an overview). In these publications, the rate of non-hematological toxicities varied between 14.6% and 45.9%, with no evidence for an increase in non-hematological toxicities with the combined modality treatment of RT and ST. In a small cohort of 39 patients, 17 of whom had received at least one novel agent, Shin et al. were unable to show increased rates of non-hematological toxicities for a combination therapy of RT and ST compared to RT alone. Only about 30% of patients developed any non-hematological toxicity, mainly dermatitis, diarrhea, or fatigue [17]. Similar results were shown by Salgado et al. in a cohort of 130 patients, with no significant difference in the occurrence of acute (within four weeks) and subacute (within six months) non-hematological toxicities [18]. In accordance, Guerini et al. failed to reveal significant differences in acute non-hematological side effects (during RT, one month after, and three months after) between RT alone, conventional system therapy, and ST with novel agents in a cohort of 312 patients. In relation to the number of RT series, non-hematological side effects occurred in 41% of cases during RT, which were primarily low-grade (grade I or II) [19].

Regarding radiation treatment, various RT fractionation regimens have been postulated in the literature, differing between a single 8 Gy fraction, hypofractionated regimes, and up to 50 Gy in normofractionation [32,33,34,35]. Comparing these fractionations, previous studies could elaborate on the superiority of a hypofractionated (30 Gy in 10 fractions) RT treatment compared to 8 Gy in one fraction concerning quality of life [33]. Higher doses have been correlated with significantly better pain relief [34,36] and motor function gain after spinal cord compression [35]. Guidelines from the International Lymphoma Radiation Oncology Group (ILROG) recommend the use of hypofractionated regimens with a dose of 8 to 30 Gy for osseous lesions, whereby a single 8 Gy fraction should be considered in patients with poor prognosis [32]. Prompted by the COVID-19 pandemic and limited treatment resources [16], recent publications point towards the use of low-dose RT regimens as a feasible treatment alternative. In a retrospective analysis, Price et al. compared low-dose radiation with 12 Gy or less to higher doses and found no significant differences in the duration of analgetic response [37]. A prospective multi-institutional study by the ILROG investigating the use of a 2 × 2 Gy regimen is currently in progress [38].

In this regard, the total dose used in the current analysis is high to achieve optimal recalcification and stabilization. This is supported by Stölting et al., who were able to show a significant increase in recalcification of osteolytic lesions using doses of ≥50 Gy compared to <30 Gy in a cohort of 138 patients [34]. Similar results were shown by Matuschek et al. in a cohort of 69 patients treated with doses of 20 to 60 Gy, in which the use of higher total doses led to a significantly higher rate of recalcification [36]. In view of further studies, total doses of >30 Gy [6,39] also seem to lead to higher recalcification rates than hypofractionated regimens [33].

As combinations of RT and ST are essential to providing long-term responses in MM, new therapeutic agents are constantly developed. Immunotherapies with daratumumab, an anti-CD38 antibody, have been introduced as first-line therapy [28] and are combined with autologous stem cell transplantation in transplant-eligible patients. For therapy-refractory MM chimeric-antigen receptor t-cells targeted against the B-cell maturating antigen or antibodies such as teclistamab represent promising therapeutic perspectives [40,41].

This analysis bears some limitations that are inherent to its monocentric and retrospective character. Consequently, the RT and RT/ST groups were not matched, with imbalances in terms of stem cell conditioning (RT: 6.9%; RT/ST: 20.8%) and worse ECOG (11 of 12 patients with ECOG grade ≥2 in the RT/ST group). Data on comorbidities and MM immunotypes were not collected, which may impair generalization. Strikingly, the proportion of patients who received stem cell transplantation up to 3 months after completion of RT was small (8 patients, 9.8%). Although all RT indications are discussed carefully in our interdisciplinary myeloma tumor board and intended not to impair subsequent (high-dose) therapies, other studies report on a delay in stem cell mobilization and collection by RT [14]. This effect (or a selection bias) cannot be excluded beyond doubt in the present analysis. Recently developed agents like daratumumab have not been considered in our analysis but may lead to new challenges in toxicity management [28].

Additionally, variations in RT concepts and doses are limited, which restricts application to a broader group. Furthermore, no large-field conditioning concepts prior to stem cell transplantation, e.g., total body irradiation (TBI), were examined. In the past, TBI has been used in combination with melphalan but has been shown to be inferior regarding toxicity and hematological recovery, with a trend towards inferior survival in comparison to a high-dose melphalan-only conditioning strategy [42]. Modern approaches with selective targeting of bone marrow (total marrow irradiation) are now being tested in this situation [43,44].

Future studies will elaborate on new, synergistic combinations of RT and ST. With the advent of chimeric antigen receptor t-cells, RT may be implemented both as a bridging and consolidation therapy [45].

## 5. Conclusions

Radiotherapy is a safe component of the multimodal therapy of multiple myeloma. The combination of radiotherapy and systemic therapy did not lead to a significant increase in non-hematological or most hematological toxicities. The only exception was a significant increase in leukocytopenia after the completion of radiotherapy. Consequently, careful clinical and laboratory monitoring during and after radiotherapy has to be implemented. The irradiation of five or more bones led to a significant rise in thrombocytopenia and leukocytopenia during radiotherapy. In this treatment situation, a critical evaluation of the size of the target volume has to be performed, along with a discussion of the sequential radiation treatment of one/several target locations.

## Figures and Tables

**Table 1:** Patient characteristics given in absolute numbers and percentages/ranges (in parentheses).

Patient Characteristics	n (% or Range)
Number of Patients	82
Sex	
Male	50 (61)
Female	32 (39)
Median age at diagnosis, y	58.5 (39–85)
Median time to RT, m	1 (0–86)
R-ISS at initial diagnosis	
I	17 (20.7)
II	44 (53.7)
III	5 (6.1)
Not recorded	16 (19.5)
ECOG Performance Status	
0	14 (17.1)
1	27 (32.9)
2	7 (8.5)
3	5 (6.1)
4	0
Not recorded	29 (35.4)
Localization	
Spine	80 (97.6)
Pelvis	58 (70.7)
Extremities	31 (37.8)
Skull	32 (39.0)
Rib cage	35 (42.7)
Shoulders	17 (20.7)

y: years; RT: radiotherapy; m: months; R-ISS: Revised International Staging System [22]; ECOG: Eastern Cooperative Oncology Group

**Table 2:** Treatment characteristics given in absolute numbers and percentages (in parentheses).

Treatment Characteristics	n (%)
Prior surgery	
Yes	36 (43.9)
No	46 (56.1)
Type of surgery	
Biopsy	17 (47.2)
Osteosynthesis	18 (50.0)
Laminectomy	7 (19.4)
Resection	5 (13.9)
Anti-myeloma agents applied within 30 days prior to 90 days after RT *	
PI	56 (68.3)
IMiD	32 (39.0)
Glucocorticoid	72 (87.8)
Alkylating Agent	35 (42.7)
Topoisomerase Inhibitor	16 (19.5)
Others	4 (4.9)
Anti-myeloma agents within concurrent systemic therapy **	
PI	39 (47.6)
IMiD	16 (19.5)
Glucocorticoid	53 (64.6)
Alkylating Agent	12 (14.6)
Topoisomerase Inhibitor	2 (2.4)
Others	1 (1.2)
Time range of systemic therapy ***	
Up to 30 d before	39 (47.6)
30 d before	10 (12.2)
14 d before	22 (26.8)
7 d before	31 (37.8)
<7 d before	36 (43.9)
During	53 (64.6)
Up to 90 d after	75 (91.5)
14 d after	61 (74.4)
30 d after	67 (81.7)
90 d after	60 (73.2)
Stem Cell Mobilization	13 (15.9)
Auto-SCT	8 (9.8)
Osteoprotection	68 (82.9)

* systemic therapy in a period of 30 days before to 90 days after radiotherapy; ** systemic therapy during RT; *** relative to RT; RT: radiotherapy; PI: proteasome inhibitors; IMiD: immunomodulatory imide drugs; d: days; Auto-SCT: autologous stem cell transplantation.

**Table 3:** Radiotherapy characteristics given in absolute numbers and percentages/ranges (in parentheses).

Radiotherapy Characteristics	n (% of PTV or Range)
PTV	
Number	134
Per patient	1 (1–4)
Irradiated bones per patient	5 (1–14)
Dose-category	
<20 Gy	1 (0.7)
20-40 Gy	75 (56.0)
>40 Gy	51 (38.1)
Not recorded	7 (5.2)
Fractions	20 (4–33)
Dose per Fraction	2 Gy (1.8–3 Gy)
Technique	
2D	8 (6.0)
3D-CRT	54 (40.3)
IMRT, Sliding-Window-technique	41 (30.6)
IMRT, VMAT	18 (13.4)
Tomotherapy	3 (2.2)
Not recorded	10 (7.5)
Localization *	
Spine	95 (70.9)
Pelvis	34 (25.4)
Extremities	3 (2.2)
Skull	2 (1.5)
Ribcage	6 (4.5)
Shoulder	3 (2.2)

* Multiple answers were possible for each patient; Gy: Gray; PTV: planning target volume; 3D-CRT: three-dimensional conformal radiation therapy; IMRT: intensity modulated radiation therapy; VMAT: volumetric modulated arc therapy.

**Table 4:** Non-Hematological toxicities given in absolute numbers and percentages (in parentheses)**.** Grading based on the National Cancer Institute’s Common Terminology Criteria for Adverse Events version 5.0 (CTCAE) [21]. Low-grade toxicities were defined as grade I or II adverse events; high-grade refers to grade III or IV. No grade V toxicities (death) were observed. For each patient, only the severest grade of toxicity from the respective category was considered.

Non-Hematological Toxicity and Grade	Total (n = 82)	RT (n = 29)	RT/ST (n = 53)	*p*
Any Non-Hematological Toxicity *				
Any grade	54 (65.9)	19 (65.5)	35 (66)	1.000
Low-grade	50 (61.0)	19 (65.5)	31 (58.5)	0.638
High-grade	14 (17.1)	6 (20.7)	8 (15.1)	0.550
Fatigue				0.735
Low-grade	18 (22.0)	6 (20.7)	12 (22.6)	
High-grade	1 (1.2)	0	1 (1.9)	
Skin				0.718
Low-grade	27 (32.9)	9 (31.0)	18 (34.0)	
High-grade	1 (1.2)	0	1 (1.9)	
Gastrointestinal				0.251
Low-grade	11 (13.4)	2 (6.9)	9 (17.0)	
High-grade	3 (3.7)	2 (6.9)	1 (1.9)	
Mucosa				0.814
Low-grade	11 (13.4)	4 (13.8)	7 (13.2)	
High-grade	4 (4.9)	2 (6.9)	2 (3.8)	
Nausea				1.000
Low-grade	10 (12.2)	3 (10.3)	7 (13.2)	
High-grade	0	0	0	
Vomiting				1.000
Low-grade	2 (2.4)	1 (3.4)	1 (1.9)	
High-grade	0	0	0	
Pulmonary				0.524
Low-grade	2 (2.4)	1 (3.4)	1 (1.9)	
High-grade	2 (2.4)	0	2 (3.8)	
Neurologic				0.233
Low-grade	4 (4.9)	0	4 (7.5)	
High-grade	1 (1.2)	0	1 (1.9)	
Musculoskeletal				1.000
Low-grade	1 (1.2)	0	1 (1.9)	
High-grade	0	0	0	
Other				0.819
Low-grade	6 (7.3)	2 (6.9)	4 (7.5)	
High-grade	4 (4.9)	2 (6.9)	2 (3.8)	

* Referring to the number of patients who suffered at least one non-hematological toxicity of any grade, low-grade or high-grade.

**Table 5:** Hematological toxicities given in absolute numbers and percentages (in parentheses)**.** Grading based on the National Cancer Institute’s Common Terminology Criteria for Adverse Events version 5.0 (CTCAE) [21]. Information on hematological toxicities was available for a variable number of patients depending on the accessibility of laboratory values. Percentage values are adjusted relative to the basic population.

Hematological Toxicity	Time	Grade	Total	RT	RT/ST	*p*
	Before RT	No Toxicity	15 (18.3)	5 (17.2)	10 (18.9)	0.982
		Low-Grade	42 (51.2)	13 (44.8)	29 (54.7)	
		High-Grade	6 (7.3)	2 (6.9)	4 (7.5)	
		Not recorded	19 (23.2)	9 (31.0)	10 (18.9)	
Anemia	During RT	No Toxicity	5 (6.1)	2 (6.9)	3 (5.7)	0.474
		Low-Grade	50 (61.0)	16 (55.2)	34 (64.2)	
		High-Grade	8 (9.8)	1 (3.4)	7 (13.2)	
		Not recorded	19 (23.2)	10 (34.5)	9 (17.0)	
	After RT	No Toxicity	2 (2.4)	1 (3.4)	1 (1.9)	0.532
		Low-Grade	55 (67.1)	18 (62.1)	37 (69.8)	
		High-Grade	11 (13.4)	2 (6.9)	9 (17.0)	
		Not recorded	14 (17.1)	8 (27.6)	6 (11.3)	
	Before RT	No Toxicity	50 (61.0)	13 (44.8)	37 (69.8)	0.255
		Low-Grade	10 (12.2)	5 (17.2)	5 (9.4)	
		High-Grade	1 (1.2)	0	1 (1.9)	
		Not recorded	21 (25.6)	11 (37.9)	10 (18.9)	
Thrombocytopenia	During RT	No Toxicity	33 (40.2)	12 (41.4)	21 (39.6)	0.184
		Low-Grade	26 (31.7)	6 (20.7)	20 (37.7)	
		High-Grade	1 (1.2)	1 (3.4)	0	
		Not recorded	22 (26.8)	10 (34.5)	12 (22.6)	
	After RT	No Toxicity	14 (17.1)	8 (27.6)	6 (11.3)	0.063
		Low-Grade	38 (46.3)	9 (31.0)	29 (54.7)	
		High-Grade	15 (18.3)	4 (13.8)	11 (20.8)	
		Not recorded	15 (18.3)	8 (27.6)	7 (13.2)	
	Before RT	No Toxicity	47 (57.3)	14 (48.3)	33 (62.3)	0.806
		Low-Grade	13 (15.9)	4 (13.8)	9 (17.0)	
		High-Grade	1 (1.2)	0	1 (1.9)	
		Not recorded	21 (25.6)	11 (37.9)	10 (18.9)	
Leukocytopenia	During RT	No Toxicity	22 (26.8)	6 (20.7)	16 (30.2)	0.684
		Low-Grade	31 (37.8)	11 (37.9)	20 (37.7)	
		High-Grade	9 (11.0)	2 (6.9)	7 (13.2)	
		Not recorded	20 (24.4)	10 (34.5)	10 (18.9)	
	After RT	No Toxicity	18 (22.0)	7 (24.1)	11 (20.8)	0.042
		Low-Grade	22 (26.8)	10 (34.5)	12 (22.6)	
		High-Grade	28 (34.1)	4 (13.8)	24 (45.3)	
		Not recorded	14 (17.1)	8 (27.6)	6 (11.3)	

**Table 6:** Overview of studies on the tolerability of concurrent radiotherapy and systemic therapy. Of note, the definition of concurrent systemic therapy differed.

Study	n	Median FU (m)	Dose (Gy)	IMRT * (%)	Novel Agent (%)	Non-Hematological Toxicity (Any grade)	Hematological Toxicity (Grade III or IV)
Shin et al., 2014 [17]	39	6	8–37.5 (Mean 26.8)	n.a.	43.6%	23.8–40% **	n.a.
Salgado et al., 2019 [18]	130	14	2–40 (Median 20)	15.4%	70%	45.9%	n.a.
Guerini et al., 2022 [19]	312	n.a.	8–30	3.5%	48.7%	41%	n.a.
Nehlsen et al., 2022 [20]	55	59.8	8–32.5 (Median 20)	n.a.	40%	14.6%	35%
Present study	82	46.5	8–59 (Median 40)	46.3%	85.4%	65.9%	45.1%

* in relation to the number of planning target volumes ** percentages differed depending on the subgroup analyzed; n: number of patients; FU: follow-up; m: months IMRT: intensity-modulated radiation therapy; Gy: Gray; n.a.: not available.

## Data Availability

Data relevant to this study are presented in the paper. Public deposition of data is not possible due to restrictions put in place by the Institutional Review Board.

## Supplementary Materials

The following supporting information can be downloaded at: https://www.mdpi.com/article/10.3390/cancers15112909/s1, Table S1: Any Hematological Toxicity.
