# Supplementary material for: Radiotherapy in Combination with Systemic Therapy for Multiple Myeloma—A Critical Toxicity Evaluation in the Modern Treatment Era
Source: Cancers (Basel). 2023 May 25;15(11):2909. doi: 10.3390/cancers15112909 (PMC10251922; doi:10.3390/cancers15112909)
Supplement: Supplementary file 1 [file cancers-15-02909-s001.zip › cancers-2390614-supplementary.pdf]

Table S1. Hematological toxicities given in absolute numbers and percentages (in parentheses). Grading based on the National Cancer Institute's Common Terminology Criteria for Adverse Events version 5.0 (CTCAE) [21]. Information on hematological toxicities were available for a variable number of patients depending on accessibility of laboratory values. Percentage values are adjusted relative to the basic population.

Hematological Toxicity and Grade	Total	RT	RT/ST	p
Any hematological toxicity: Entire period*				
Low-Grade	73 (89)	23 (79.3)	50 (94.3)	
Not recorded	9 (11)	6 (20.7)	3 (5.7)	
High-Grade	37 (45.1)	8 (27.6)	29 (54.7)	0.349
Not recorded	25 (30.5)	14 (48.3)	11 (20.8)	
Any hematological toxicity: Before RT*				
Low-Grade	46 (56.1)	14 (48.3)	32 (60.4)	1.000
Not recorded	20 (24.4)	10 (34.5)	10 (18.9)	
High-Grade	7 (8.5)	2 (6.9)	5 (9.4)	1.000
Not recorded	20 (24.4)	10 (34.5)	10 (18.9)	
Any hematological toxicity: During RT *				
Low-Grade	59 (72.0)	18 (62.1)	41 (77.4)	1.000
Not recorded	19 (23.2)	10 (34.5)	9 (17.0)	
High-Grade	16 (19.5)	4 (13.8)	12 (22.6)	0.754
Not recorded	22 (26.8)	10 (34.5)	12 (22.6)	
Any hematological toxicity: After RT *				
Low-Grade	61 (74.4)	18 (62.1)	43 (81.1)	0.668
Not recorded	14 (17.1)	8 (27.6)	6 (11.3)	
High-Grade	31 (37.8)	5 (17.2)	26 (49.1)	0.018
Not recorded	15 (18.3)	8 (27.6)	7 (13.2)	

* Patients can be listed both in the high- and low-grade group due to the combination of several categories.
